# Participatory design research for the development of real-time simulation models in healthcare

**DOI:** 10.1080/20476965.2023.2175730

**Published:** 2023-02-09

**Authors:** Alison Harper, Navonil Mustafee

**Affiliations:** a PenCHORD, University of Exeter Medical School, Exeter, United Kingdom; b Centre for Simulation, Analytics and Modelling (CSAM), University of Exeter Business School, Exeter, United Kingdom

**Keywords:** Design science research, participatory modelling and simulation, real-time simulation

## Abstract

The implementation challenges for modelling and simulation in health and social care are well-known and understood. Yet increasing availability of data and a better understanding of the value of Operational Research (OR) applications are strengthening opportunities to support healthcare delivery. Participative approaches in healthcare modelling have shown value through stakeholder engagement and commitment towards co-creation of models and knowledge but are limited in focus on model design and development. For simulation modelling, a participative design research methodology can support development for sustained use, emphasising model usefulness and usability using iterative cycles of development and evaluation. Within a structured methodology, measures of success are built into the design process, focusing on factors which contribute to success, with implicit goals of implementation and improvement. We illustrate this through a participative case study which demonstrates development of the component parts of a real-time simulation model aimed at reducing emergency department crowding.

## 1. Introduction

Health and social care services have been under strain for decades (e.g., Amalberti & Vincent, 2020), and despite the rapid and innovative pivots in health service delivery and planning as a result of the recent global pandemic, substantial disruption continues worldwide (Arsenault et al., 2022). With increasing access to data and demand for data-driven decision-support, OR researchers are therefore challenged to further develop and integrate useful, usable, and sustainable decision-support tools to benefit under-resourced healthcare organisations and the populations they serve. Real-time simulation models are initialised and driven by real-time (or near real-time) data and can provide much needed information to support decision-making in systems that continuously make decisions in real-time. The outputs of the simulation aim to guide constrained and enabled safe action, and design, development and evaluation choices can influence whether these are as intended. Such models are designed for recurrent-use and combine usability features with technical aspects, adding complexity to model design. To explore the complex challenges addressed by design, it may be necessary for modelling and simulation researchers to participate in development and design processes using participatory modes of practice.

A participatory approach to modelling through enhanced stakeholder engagement conveys a collective understanding of modelling, sharing knowledge, and policy design (Adams et al., 2022). Participative practice is an approach to research which incorporates local knowledge and collaborative activities in an iterative, flexible design (Cornwall & Jewkes, 1995). Through collaborative working, participatory research practices in OR such as *facilitated modelling* have been driven by an interest in supporting decision-makers who are engaged with complex problems (Franco & Montibeller, 2010; Franco, 2013). Similarly, participative methodologies for quantitative modelling and simulation (M&S), such as *PartiSim* (Kotiadis & Tako, 2016, 2018; Tako & Kotiadis, 2015), *SimLean* (Robinson et al., 2012) and *Simtegr8* (Tako et al., 2019) have demonstrated successful M&S practice with a focus on implementation and change. Modelling based on local, contextual knowledge can cut through knowledge controversy, for example Harper et al. (2021) viewed M&S participatory practice through the lens of social learning, lowering the risk of misrepresenting the goals and values of stakeholders.

It is widely accepted that effective stakeholder engagement influences the outcomes of a modelling study in a social organisation and is more likely to support acceptance and implementation of model results (e.g., Long & Meadows, 2018), yet De Gooyert et al. (2017) reported a lack of attention towards implementation and results in OR studies. As healthcare organisations continue to be both under pressure, and reactive in their decision-making (Sujan et al., 2021), participative methodologies that require substantial engagement time from healthcare staff present significant challenges for applied M&S researchers. Additionally, while facilitated modelling and other participatory approaches support problem understanding and solution generation, they can be limited in their ability to underpin formal model development. This is particularly the case where the model is designed for recurrent-use, requiring iterative development and testing of component parts. For real-time simulation, technical requirements include data acquisition, data analysis, integration, scenario management, the simulation model, and appropriate outputs (Onggo et al., 2021). Additional design issues require close consideration such as usability, safety, and potential unintended uses of the model, its outputs and its user documentation. These are challenging endeavours in any system, with healthcare bringing additional barriers such as stringent data governance structures.

Design Research (DR) involves the collaborative building and evaluation of artefacts, such as models, designed to meet identified business needs (Hevner et al., 2004). The approach is flexible, iterative and participative (van Oorschot et al., 2022). It is focused on developing a solution towards solving a problem yet has rarely been utilised in OR as a research strategy for supporting M&S studies. This paper proposes the use of DR for supporting participative M&S studies for simulation model design, development, use and maintenance in healthcare. The contribution of this paper is demonstrating the application and value of a DR approach towards the development of the constituent parts of a real-time simulation model in an emergency department in a UK-based health service. These constitute real-time data feeds, forecasting modules, and a simulation model (Mustafee et al., 2018; Onggo et al., 2018, 2021). The remainder of the paper is structured as follows. The next section describes DR methodology through a review of key literature in this area. The applicability of DR to M&S studies is discussed in Section 3. Section 4 outlines our case study using the DR approach. This is followed by section 5, which presents a critical evaluation of the opportunity for DR in relation to participative modelling. Section 6 concludes the paper, discussing the implications and opportunities for DR to support healthcare M&S delivery.

## 2. Design research: a methodology for change

Design science is a research paradigm which addresses the design of artefacts, through design research, design knowledge, and design practice. Simon (1988) distinguished between the natural sciences, concerned with explaining how things are and how they work, and design sciences which are concerned with how to design artificial artefacts with desired properties. These may be to solve a problem, create change, or improve an existing solution (Baskerville et al., 2015). Artefacts are defined as “any designed object in which a research contribution is embedded in the design” (Peffers et al., 2007). This may be a construct, model or method (Hevner et al., 2004), with progressive refinements studied in target settings. Articulation of principles that underpin its impact support wider applicability (Van den Akker et al., 2006).

Within design science, design research (DR) is widely used across a range of applied research disciplines, including engineering, computer science and information systems (IS), bridging theory and practice. Different disciplines have distinctly different design goals, although methodologies share a core of common activities and follow a similar iterative, step-wise design (Gericke & Blessing, 2012). Peffers et al. (2006, 2007) investigated DR process elements across disciplines to draw out common elements towards an IS DR process, finding the following core activities: (i) Identify problem and motivation; (ii) Define objective of a solution; (iii) Design and development; (iv) Demonstration; (v) Evaluation; (vi) Communication. While there is a process sequence, the research may start at a number of stages depending upon the research objectives, and the process is iterative. O’keefe (2014) adapted this methodology for OR, adding theory generation as a final stage. This asks whether the design can be used or adapted to other contexts. While this is important for researchers who aim to generalise their work, Peffers et al. (2007) described the DR methodology as “solution-oriented”, emphasising the analysis of the design idea, rather than “problem-oriented”, focusing on the analysis of the addressed problem.

**Figure 1.**
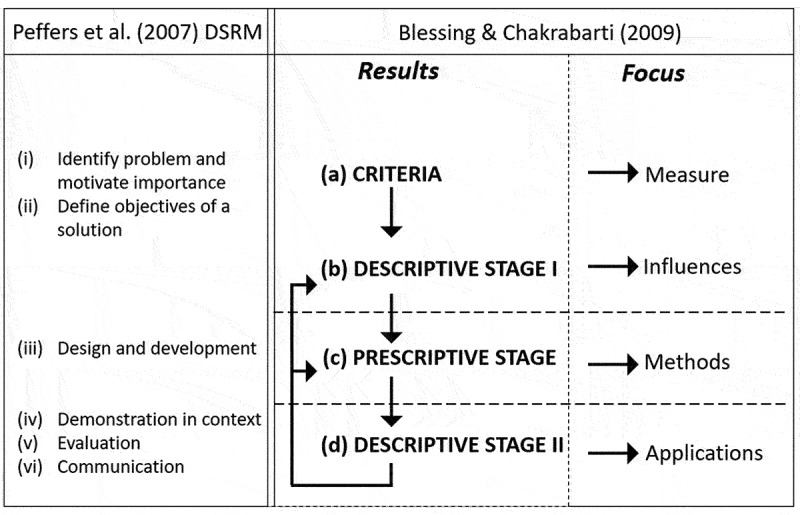
DR methodology frameworks adapted from Peffers et al. (2007) and Blessing and Chakrabarti (2009).

For many OR situations, a solution-oriented approach is appropriate. However, where problems are complex, changing, conflicting, contingent and partly incommensurable, an applied approach situated within the problem context is required (Ulrich, 2012), in contrast to allowing methods to dictate problem definitions. Participatory practice is requisite, and is well-embedded in DR and practice (van Oorschot et al., 2022). In contrast to the methodology proposed by Peffers et al. (2007), the DR methodology outlined by Blessing and Chakrabarti (2009) explicitly focuses on understanding and unfolding the problem situation. They emphasised the need to identify criteria and success measures for evaluation of the intervention; design factors that may influence these criteria; and how to capture knowledge gained from the design process to develop guidelines, methods and tools that can support the development of future similar artefacts in similar domains. Figure 1 illustrates these stages, mapped to the activities proposed by Peffers et al. (2007):

- Determining the key criteria for evaluating the intervention, defining the specific research problem, justifying the value of the solution, and then inferring the objectives of a solution from the problem definition and knowledge of what is possible and feasible.
- Identifying the influences on evaluation criteria, how these influences interact, and how they can be measured i.e., how to improve the design process for the context.
- Artefact development determines the functionality, its architecture, and develops the artefact based on knowledge of theory and other information sources.
- Evaluation involves understanding how the knowledge gained from the design process can be used to develop guidelines, methods and tools, and how this design support can be evaluated. Evaluation is needed to determine how to improve the chances of developing a plausible intervention as determined by the criteria in Stage (a). Peffers et al. (2007) encompass this principle through demonstration of the use of the artefact towards solving the problem, and evaluation of how well the artefact supports a solution to the problem, using appropriate methods. The final stage is communication of the problem and its importance; the artefact and its utility, novelty, rigour of design; and effectiveness of its approach to appropriate audiences in practice and academia. This might support progressing the design process, applying it to another research domain, or using it to solve a different problem.

## 3. Design research for M&S

**Figure 2.**
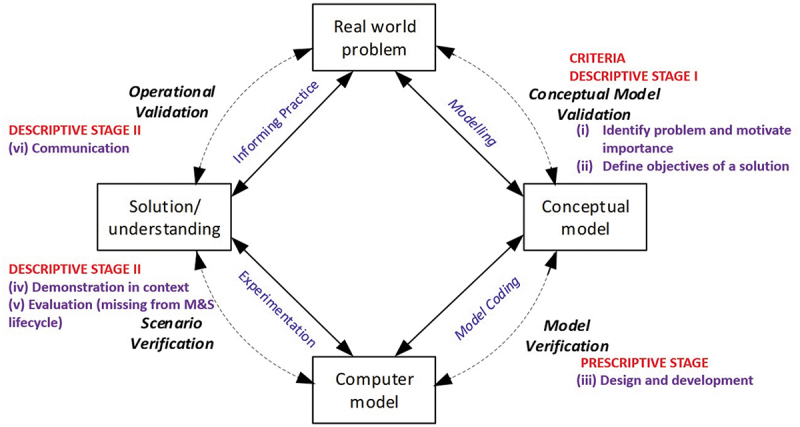
M&S lifecycle, adapted from Brooks and Robinson (2000) and Sargent (2004).

The DR process supplements the M&S lifecycle [Figure 2, mapped with DR stages defined by Peffers et al. (2007) and Blessing and Chakrabarti (2009)] by incorporating an evaluation component. This means that measures of success are built into the design process, and model development is focussed on factors which contribute to success. Additionally, the DR process is intrinsically participative across all stages (Peffers et al., 2018; van Oorschot et al., 2022).

Any of the stages of a DR methodology may be the focus of an individual project. It may be possible to build on existing research, or the process may involve one or more empirical studies. The stages from Blessing and Chakrabarti (2009) are discussed in relation to the M&S lifecycle:

- **Criteria Definition**

This stage involves a clarification of the research by reviewing the literature and collaborating with stakeholders to determine the aim, focus and scope of the research project, and how the findings can be used to improve design. It is then possible to determine the factors that have a negative or positive influence on a plausible solution to enable evaluation of the developed artefact in *Descriptive Stage II*. In the M&S lifecycle, this equates to problem definition. However, as many M&S studies do not aim to build an enduring artefact, the M&S problem definition stage does not consider evaluation criteria. To be problem-focussed, a participative approach can establish the “why” of the study through success criteria.

- **Descriptive Stage I**

Having identified the criteria for success, an understanding of the factors that influence, directly or indirectly, the above criteria focus the modelling process and its evaluation on factors which contribute to success. These are derived from the literature as theoretical propositions (Carlsson, 2006), from site visits, direct observation (McKenney & Reeves, 2018), workshops (Blessing & Chakrabarti, 2009), or other methods such as interviews or questionnaires (Salehi & McMahon, 2009). This stage aligns with M&S conceptual modelling, which may utilise similar methods, but will also translate the most important success criteria into measurable criteria, which may be quantitative and/or qualitative. For M&S, quantitative criteria are essential, for example assuring data quality and model validation. However qualitative data are also important, as a technically “correct” model may still fail to inform or be integrated into practice (Jahangirian et al., 2017).

- **Prescriptive Stage**

This involves determining the functionality and architecture, and developing or prototyping the model and/or other artefacts (McKenney & Reeves, 2018), aligning with the stage of computer model development in the M&S lifecycle, including validation/verification activities. A single study may focus on one or more parts of this process, or one or more iterations (Blessing & Chakrabarti, 2009). This stage links with success criteria, so elements such as documentation clarity and usability, user interface design, output automation and visualisation, handover workshops or presentations etc. may also be considered as part of the overall purpose of the study. As participatory practices, lack of participation and mutual engagement in the research process will reduce the likelihood of a useful outcome (Madsen & O’mullan, 2018).

- **Descriptive Stage II**

The second descriptive stage is evaluation, undertaken to determine whether the model has the expected effect on influencing factors identified in *Descriptive Stage I*. It evaluates the functionality of the model or tool from the user perspective and provides feedback for further development. This stage is not represented in the M&S lifecycle, and not all M&S studies will require it. However, for real-time simulation models, which require access to real-time system data and are designed for recurrent decision-support, this stage can support the sustainable transfer from academia to practice. Evaluation requires questions about usefulness, implications and unintended consequences, and is best achieved in context. It should look to investigate desired and undesired effects, direct and indirect effects and immediate and long-term impacts; and to account for users and situational context. At the end of this activity the researchers can decide whether to iterate back to a previous activity to try to improve the effectiveness of the model/artefact or to leave further improvement to subsequent projects.

## 4. Case study: real-time simulation modelling for short-term decision-support in an emergency department

The following case study demonstrates the value of DR for a real-time simulation study in an emergency department (ED) aimed at reducing crowding. Multiple iterations were undertaken towards the development of the constituent parts of the system: real-time data feeds, forecasting modules, and a simulation model with scenario management. These iterations are synthesised and presented as one cycle in the following overview. One or more further iterations, which are work-in-progress, will result in integration of the components. A real-time simulation constitutes a data acquisition system with a validated simulation model and other methods of data analysis, such that the simulation model is initialised using real-time system data (Mustafee et al., 2018), while decisions taken as a result of simulation outputs will subsequently impact the system and be reflected in system data. Real-time simulations may therefore be termed “symbiotic simulation” (Onggo et al., 2021).

The questions of interest to the case study hospital were: (i) whether real-time and forecasted wait-times are useful and safe for patient decision-making, and (ii) whether forecasted crowding and redirecting non-urgent patients to alternative treatment centres can support capacity planning for ED given predicted high patient numbers. Levin et al. (2012) reported growing evidence of a relationship between ED crowding and patient safety, where the system decompensates, that is, exhausts its capacity to adapt. Staff manage pressures by making in situ adaptations and goal trade-offs, but this requires awareness of the situation to respond in an appropriate and timely way, which real-time simulation can provide. In this case, the real-time data provides information across the urgent care system in four facilities: ED and three alternative minor treatment centres.

**Figure 3.**
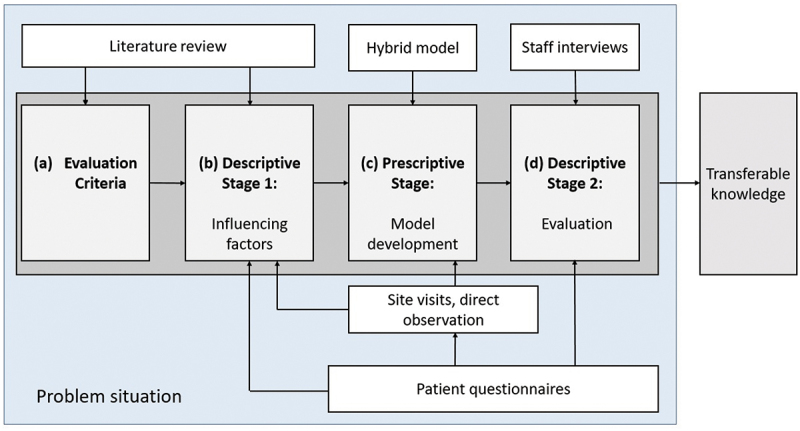
Design Research Methodology (from Blessing & Chakrabarti, 2009) mapped to methods used in the case study application.

Having identified the problem, the criteria were defined from the literature. DS Stage I was derived from site visits, workshops, direct observations, and patient questionnaires. The Prescriptive Stage developed a prototype model combining real-time wait-time data, forecasted wait-times, and a discrete-event simulation model (DES). DS Stage II evaluated this iteration of the model components using staff interviews. This process is illustrated in Figure 3.

### 4.1. Stage 1: evaluation criteria

The criteria for evaluation looked at a wide set of issues derived from the literature. The overall criterion for success was usefulness for short-term decision-support (Jahangirian et al., 2012). Other factors included safety (McGeorge et al., 2015; Peute et al., 2013), efficacy (Brailsford et al., 2013), and cost-effectiveness of the application; perceptions of the usability (Berggren et al., 2011; Endsley, 2016) and functionality of the model (Weiner et al., 2016); confidence in the real-time applications to provide short-term decision support, including its reliability and accuracy (Sanjay & Allamma, 2016); the degree to which the model fits into staff workflow (Brailsford et al., 2013); and model maintenance and sustainability (Greenhalgh et al., 2017). As this was exploratory work and early in the design process, each of these were retained as measurable criteria to be evaluated qualitatively in Stage 4.

### 4.2. Stage 2: descriptive study I

The application centred on two audiences: healthcare providers (staff), and healthcare users (patients). For staff, an understanding of influencing factors were supported through site visits and workshops (Table 1):

**Table 1.** Staff engagement activities.

Activity	Researcher	Participants	Number
Site visits and direct observations	Primary researchers	Collaborating NHS staff: senior management, senior clinical staff, ED operations manager, IT/analyst staff, ED site visits and hospital control room	Approximately 25 visits were undertaken, including ED walk-through
Workshops	Primary researchers and other academic staff	(i) One-day network event with academics; NHS management, IT, clinical and communications staff; patient representatives; and academics [21/06/2016; University of Exeter] (ii) Two mapping events with academic, clinical and NHS management staff [10/07/2018; 27–28/06/2019 - both workshops conducted in an NHS Trust in the South West of England]	(i) 39 participants + 9 academics (ii) [a] 7 participants (ii) [b] 7 participants (5 in common with previous event)

Workshops were recorded and thematically summarised. Staff were primarily concerned about patient safety, and their inadequate understanding of why patients with low-acuity conditions choose to attend ED. If successful, they agreed the application would support joint working between providers; empower, educate, and inform patients; improve resource utilisation across the network by spreading demand; reduce anxiety in patients; and reduce patient waits. Staff saw value in the use of real-time and forecasted patient numbers for decision-support to improve patient flow and adaptive behaviours, and were interested in a simulation model that could support system recovery, given a predicted high number of patient arrivals in a 2–4 hour time period. This was seen to support ED operational targets and patient choice, but there remained a general concern for the risk of a suboptimal outcome for patients, who either chose, or were redirected to a low-urgency facility.

As patient safety was of primary concern, a literature review was undertaken to obtain an understanding of the various factors that influence, directly or indirectly, safety. A range of non-urgent ED attendance reasons have been well-documented in the literature (e.g., Chapman & Turnbull, 2016; Cheek et al., 2016; Krebs et al., 2017; Weber et al., 2017), but knowledge of wait-times had not been previously investigated. For this reason, a descriptive study was undertaken to determine whether knowledge of real-time and forecasted wait-times would influence patient attendance decisions, and the acceptability of being re-directed or choosing to attend a different care facility at busy times.

A patient questionnaire examined patient attendance decisions. It was developed from a purposeful review of the literature regarding factors influencing attendance decisions and involved a convenience sample of 152 low-acuity patients waiting for emergency care (Harper, 2021). *Low acuity* was defined by the assignment of priority Triage Category 4 or 5, and that patients walked-in, that is, were not transported by ambulance. The results of the questionnaire indicated that real-time data applications have the potential to contribute to reducing emergency crowding by influencing patient health-seeking behaviour, in particular in younger, anxious patients who are in better health. Patients saw the benefit at the system-level of spreading demand across an urgent-care network, and of lower-acuity patients using more appropriate facilities.

### 4.3. Stage 3: prescriptive study

**Figure 4.**
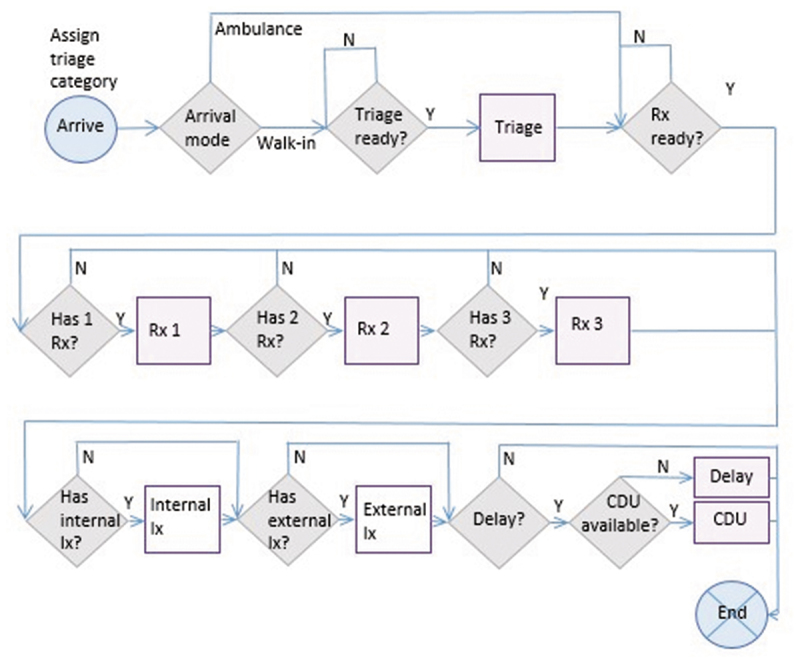
Flowchart of ED processes for DES. Rx=Treatment; Ix=Investigation; CDU=Clinical Decision Unit (clock stops). Delay=delay to discharge (e.g., awaiting admission, transport etc.).

**Figure 5.**
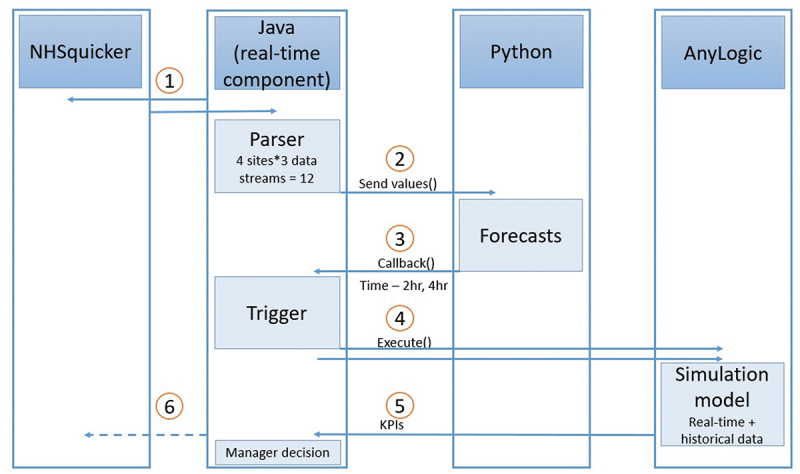
Planned integration of component parts.

This stage involves model artefact development. The outcome of the descriptive study was used to inform the design of a hybrid model (Powell & Mustafee, 2017). The model was developed in AnyLogic (Harper, 2021) and validated in four parts: the *descriptive* component (identifying the data requirements and availability), the *diagnostic* component (identifying a trigger for the simulation model), the *predictive* component (developing a forecast model for a forecasted trigger in Python) and the *prescriptive* component (a validated simulation model, using mixed real-time initial conditions and a warm-up period). The objective of the prescriptive studies was to develop the constituent artefacts for a real-time simulation model towards a real-time integrated system. The components are executed separately and the integration between components is work-in-progress. Real-time data was made available by the *NHSquicker* platform, of which one component is a mobile phone application which provides real-time wait-time data for patients across the southwest of England with the aim of supporting attendance decisions (Mustafee & Powell, 2020; Mustafee et al., 2017). The real-time data definition, acquisition, platform development, testing and validation for *NHSquicker* was undertaken as a separate DR iteration. The forecasting model uses seasonal ARIMA to predict patient numbers up to 4-hours ahead. An hourly trigger is based on historical crowding data across a 24-hour period. Where the trigger is reached, the DES is activated to support ED recovery using scenarios including re-directing patients to alternative facilities in the urgent-care network (see Figure 5). The DES uses historical patient acuity, hourly arrival rates, proportions needing treatments/investigations, service times derived empirically, and incorporates a downstream delay, for example waiting for admission. Figure 4 illustrates the ED process diagram, represented as a flowchart.

Following a warm-up to initialise the model, the model is proposed to be updated with real-time data for the total number of patients in the department, number of patients waiting for assessment, and maximum wait-time. The model was validated by comparing model outputs with historical data from *NHSquicker* for total numbers of patients in the department, and numbers of patients waiting to be seen. Following several iterations through the DR process, the constituent parts are in place to integrate the model components into a single, automated hybrid model which updates every 30 minutes, forecasts total patients in ED, and triggers the simulation when predicted thresholds are reached (Figure 5). Integration of components is future work, further informed by the evaluation stage

### 4.4. Stage 4: descriptive study II

For this stage, semi-structured interviews were chosen for exploratory evaluation and detailed responses, started in February 2020. Participants are tabulated in Table 2. Due to Covid-19, interviews after March were curtailed, however there was significant consensus of findings across participants over approximately 6 hours of interviews.

**Table 2.** Interview participants.

Participant	Number completed (planned)
Doctors	2 (4)
Nurses and nurse practitioners	0 (2)
Executive Management	3 (4)
Data Analysts	1 (2)

The interview schedule, which guides the direction of the interview, was informed by the literature review, observations, and the patient questionnaires. It focused on the criteria identified in Descriptive Stage I as an application evaluation, assessing the functionality of the model components. The success evaluation, that is the degree to which the simulation might help to support short-term decision-support and reduce overcrowding will be tested following future development, and before the model is used in practice. Each element of the model was demonstrated separately, examining the technology, its usability, visual outputs, dependability and accuracy, and the extent to which the information generated is accepted, trusted and considered safe and sufficient for decision-support. Interview data was analysed using thematic analysis. Results indicate that while patients support demand management actions across the urgent care network, staff are consistently more focused on improving patient flow through their own system (hospital-level), and their interest in the wider system is mostly its impact on their own demand. While a simulation model of ED must incorporate downstream hospital processes, the wider network is also an important consideration when managing patient demand.

Additionally, despite the focus on hospital system activity, hospital – level challenges exist. Stakeholder engagement and management is essential for most M&S studies in a sociotechnical system, but specific challenges were identified from staff interviews. The conflicting goals and behaviours of different staff groups are likely to be a significant challenge and can impact further progress of the model development. Testing the predictive and prescriptive components will require both clinical and management support. There is often no single adoption decision, and inter-professional relationships, power and politics are important. Nonetheless, there was considerable interest in progressing the work, for example:

“Where we’re saying ‘we recognise we’ve got a problem, we need to shift our resources’, we need to understand the consequences. Because we are always moving things before we understand the consequence of it … this is absolutely why we should be using simulation”. [Doctor]

“This [the model] is going in the right direction. I’m very aware that we aren’t as data or information driven as we could be. Having data is one thing, having data which informs decision-making is something else”.[Analyst]

“I think what’s great about this, I can see the art of the possible. So if I were the COO [chief operating officer], I would be thinking I can move some of my demand around, into different places, into different pools, which we sort of inherently know, but now I can physically see the impact”.[Manager]

The evaluation identified a possible negative consequence. Both evaluation stages with staff and patients found that predicted wait-times are more likely to support attending at a different time of day, than attending a different facility. The impact of this is on the NHS, as it is better to spread demand across the system than to utilise quieter times of day in ED where staff resources are reduced.

### 4.5. Case study discussion

DR provides a flexible, rigorous methodology to support the development of simulation models for recurrent use. The case study involved several cycles of a DR methodology towards the development of the constituent parts of a real-time simulation model. The model aims to provide short-term decision-support in ED for managing variable demand and capacity within a network of facilities. Stages 1 and 2 are considered complete, while stages 3 and 4 require further iterations towards component integration and implementation. Real-world implementation and use aren’t explicit in the methodology, but are implicit in the development, evaluation and communication phases. Following the completion of several iterative cycles to develop artefact components, the evaluation stage is informing further development and integration of the simulation model, which is both contextually and more widely applicable.

The flexible approach supports contributions to practice, but also to the knowledge base, that can be generalised beyond individual solutions to problems. Offermann et al. (2011) suggest that generalisability or transferability of findings occurs where settings are similar, especially when research involves social dimensions, and insights might be transferred from one to the other. To increase the robustness of the intervention, the more situations a design has been shown to work, the more likely it is considered to work for similar new problems. For this study, as well as informing future development in the case study site, findings from both descriptive stages can be generalised towards the development of similar components for real-time models with both patient and staff applications in other ED departments. The approach can additionally offer value in other systems where short-term decision-support has utility, for example in social services where flexible workforce is required to deal with urgent prevention of inappropriate hospital admissions.

## 5. Opportunities for DR and participative modelling

Participatory research can increase the validity, usability and sustainability of research artefacts such as models (Allen et al., 2011). In healthcare, lack of implementation of the results of M&S studies have been a well-documented problem (e.g., Katsaliaki & Mustafee, 2011; Long et al., 2019), and participatory M&S studies have demonstrated success with sustained stakeholder engagement, a particular challenge where priorities and roles can shift rapidly. Participatory methods are recognised as offering value during M&S conceptual modelling (e.g., Lehaney & Paul, 1996; Powell & Mustafee, 2017). Extending these practices across the M&S lifecycle have resulted in improved consensus about actions to be taken by addressing learning, social, and political issues (Den Hengst et al., 2007; Proudlove et al., 2017; Tako & Kotiadis, 2015). However, real-time artefacts intended for recurrent-use benefit from co-development, capturing collective needs, interests, concerns and risks.

Using DR, measures of success are built into the design process, and model development is focussed on factors which contribute to its usability and usefulness. Additionally, unintended uses of the model or its outputs are considered, so that addressing safety and risk are part of model design. Model design is a purposeful process with functions that can enable and steer behaviour and are an outcome of choices made during design and use (Harper et al., 2022). DR can address the limitations and issues that arise during data collection, modelling processes and users’ concerns (Blessing & Chakrabarti, 2009). A related issue is that of responsibility in technology design, associated with ethics and professional codes of conduct (Herwix et al., 2022).

These concerns allow M&S studies to observe the larger system – its context, its data and where it is placed within (and beyond) the organisation. A key feature of DR is evaluation, which leads to further design, development, and evaluation. The evaluation phase has another important distinction: it is not based on the value of the underlying method or algorithm, but upon the utility or usefulness of the artefact in practice (Hevner et al., 2004). A technically more “correct” model may not have improved utility if it is not demonstrable in terms of gains that matter to stakeholders.

## 6. Conclusion

Decades of OR technical innovations have been changing the face of society, and central to these innovations is design. Yet DR has had surprisingly limited application in OR, and in M&S specifically. Royston (2013) and O’keefe (2014) both upheld the utility of design-oriented, rather than solution-focussed OR, and DR is aligned with the values and approaches of OR as an applied discipline focussing on context-based practice. M&S researchers can influence systems through simulation, scenario selection and scenario analysis. Participatory DR offers a methodological approach to healthcare simulation studies that can address design concerns which arise throughout the M&S process for improved context-based solutions. Further, while innovative M&S solutions to new problems continue to be published, the modeller’s decisions in resulting designs are often implicit, yet more explicit learning can also advance subsequent design efforts (Richey & Klein, 2014; Van den Akker et al., 2006).

This paper proposes DR as a participatory methodology which focuses on model design for recurrent-use simulation models such as real-time or near real-time simulation. The study involved both patient and staff stakeholders to support an integrative view of healthcare service delivery. It provides an illustrative case study that demonstrates the value of DR towards both contextual, and generalisable design for short-term decision-support in urgent and emergency care. Several studies have investigated how to innovate and improve the use of real-time simulation in healthcare (e.g., Augusto et al., 2018; Oakley et al., 2020). However, if sustained model use and real-world change are to be achieved, there is a need in parallel to manage design, usability and risk to ensure that the satisfaction and safety of users is not compromised. DR investigates desired and undesired, direct and indirect, and short- and long-term effects within the situational context, and these can only be achieved using a participatory approach. Any M&S design intervention with a view to improving system functioning for healthcare delivery should aim to contend with both the technical and the social system elements. Using DR as a participatory methodology can extend the relevance, as well as the generalisability or transferability of the M&S method.
